# Neuropsychiatric lupus: a mosaic of clinical presentations

**DOI:** 10.1186/s12916-015-0269-8

**Published:** 2015-03-04

**Authors:** Shaye Kivity, Nancy Agmon-Levin, Gisele Zandman-Goddard, Joab Chapman, Yehuda Shoenfeld

**Affiliations:** The Zabludowicz Center for Autoimmune Diseases, Sheba Medical Center, Tel-Hashomer, 52621 Israel; The Dr. Pinchas Borenstein Talpiot Medical Leadership Program 2013, Sheba Medical Center, Tel-Hashomer, 52621 Israel; Department of Medicine C, Wolfson Medical Center, 61 Halochamim Street, POB 63, Holon, 58100 Israel; Department of Neurology, Sagol Neuroscience Center, Sheba Medical Center, Tel-Hashomer, 52621 Israel; Sackler Faculty of Medicine, Tel-Aviv University, 39040 Tel-Aviv, Israel; Incumbent of the Laura Schwarz-Kipp Chair for Research of Autoimmune Diseases, Tel-Aviv University, 39040 Tel-Aviv, Israel; The Zabludovicz Center for Autoimmune Diseases, The Chaim Sheba Medical Center, Tel-Hashomer, 52621 Israel

**Keywords:** Anti-ribosomal-P, Auto-antibodies, Cognitive impairment, Depression, Neuropsychiatric lupus

## Abstract

Neuropsychiatric symptoms affect nearly half of the patients with systemic lupus erythematosus; however, the effect on disease severity, quality of life, and prognosis is tremendous. Symptoms of neuropsychiatric systemic lupus erythematosus may range from mild diffuse ones, to acute life threatening events. Although the underlying mechanisms are still largely unraveled, several pathogenic pathways are identified, such as antibody-mediated neurotoxicity, vasculopathy due to anti-phospholipid antibodies and other mechanisms, and cytokine-induced neurotoxicity. In the current review, we describe the old and the new regarding epidemiology, pathophysiology, diagnosis, and management of neuropsychiatric systemic lupus erythematosus. The possible link between neuropsychiatric symptoms and specific mechanisms may help to facilitate our understanding of the disease in the future, thus allowing for better treatment strategies.

## Introduction

Patients with systemic lupus erythematous (SLE) that suffer from one or more of several neuropsychiatric symptoms represent a subcategory termed ‘neuropsychiatric lupus’ (NPSLE). Cohorts of SLE patients suggest that nearly half will suffer from NPSLE during their disease course. The definition of NPSLE is a tough challenge owing to the broad spectrum of neuropsychiatric symptoms that it encompasses, most of which are non-specific (for example, headache, cognitive dysfunction, etc.). The most accepted effort, so far, to classify NPSLE was made by an American College of Rheumatology (ACR) expert-committee, in 1999 [1]. This committee identified 19 neuropsychiatric conditions, termed ‘case definitions’, in NPSLE patients, including 12 central nervous system (CNS) and 7 peripheral nervous system ones (Box 1). It should be noted that a few population-based studies, aimed at validating these ACR-NPSLE case definitions, did not find them to be effective in differentiating NPSLE patients from those with neuropsychiatric manifestations not associated with SLE [2]. Being a unique and complex disease, in terms of diagnoses, management, and mechanism, NPSLE merits a separate discussion. The purpose of this review is to describe the plethora of data that has accumulated during the recent years regarding NPSLE manifestations, diagnosis, and therapy.

## Review

### Epidemiology

The prevalence of SLE varies among populations and is approximately 50 in 100,000 [3]. However, the estimate of NPSLE is challenging owing to variations in study designs (prospective or retrospective), follow-up periods, the uniformity of case definitions, and disease age population evaluated (pediatric vs. adult). To overcome this obstacle, the 1999 ACR-NPSLE case definitions have been widely used; however, despite this, estimates of the prevalence of NPSLE has still ranged considerably [4]. Unterman et al*.* [5] performed a meta-analysis of studies assessing NPSLE prevalence. According to 10 high-quality prospective studies, which included 2,049 SLE patients, the prevalence of NPSLE manifestations among them was 56%. Among these approximately 90% were pure CNS manifestations. The most frequent NPSLE manifestations were headache (28.3%), mood disorders (20.7%), cognitive dysfunction (19.7%), seizures (9.9%), and cerebrovascular disease (8.0%). Epidemiological studies that excluded non-specific, minor CNS-symptoms, such as mild cognitive dysfunction, headache, mild depression, and anxiety, demonstrated a lower prevalence of NPSLE. Thus, one may suggest a new approach to defining NPSLE manifestations. This approach will address major manifestations that may serve as criteria and minor ones that are closely related to SLE but are less specific (for example, head ache, anxiety, mild memory loss, etc.).

#### The complex pathophysiology of NPSLE

**Figure 1:**
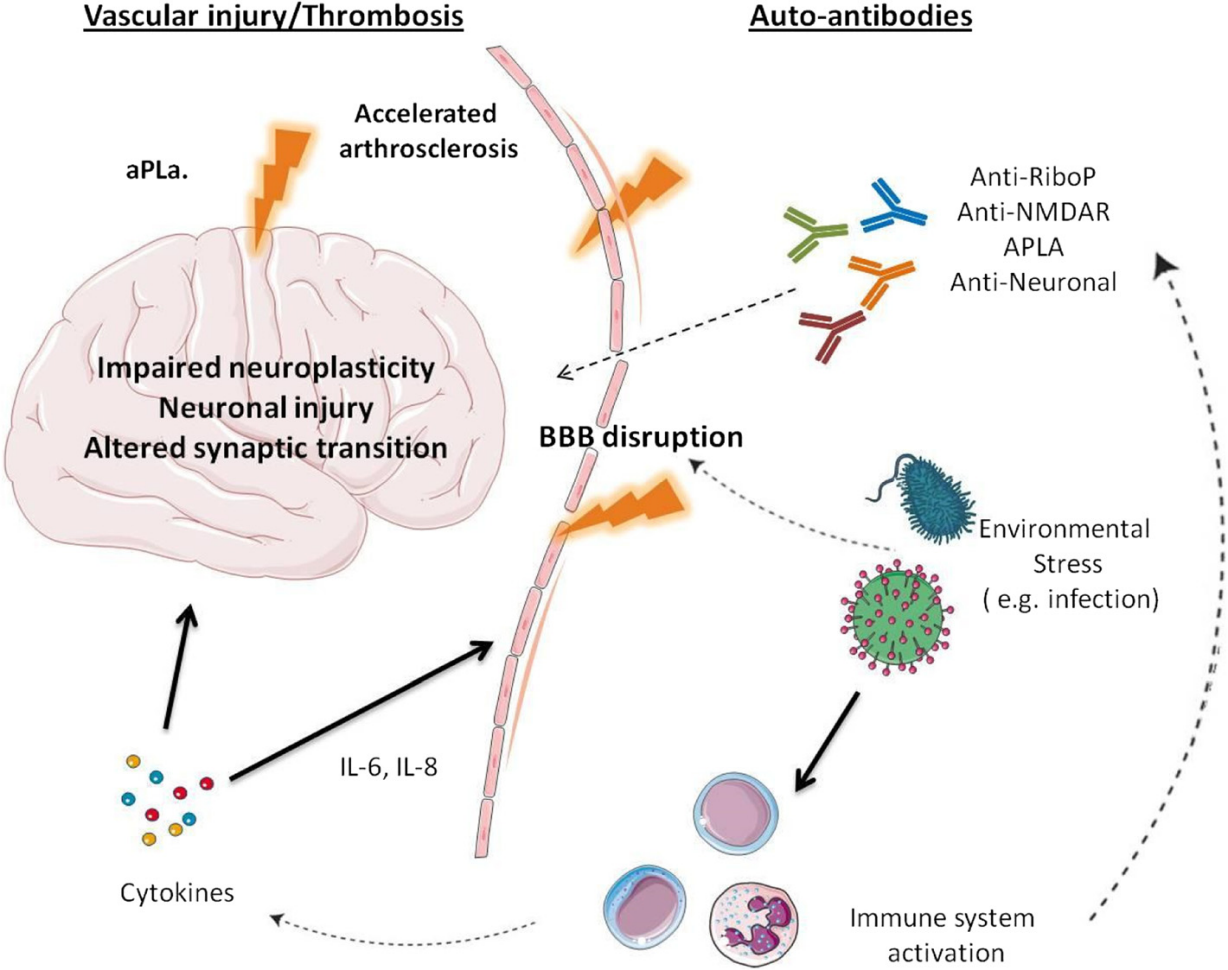
**Proposed pathogenesis of neuropsychiatric lupus.** Auto-antibodies enter the brain causing neuronal damage, including impaired neuroplasticity and synaptic transition. In order to reach the brain, the blood–brain barrier must be transiently breached by external (for example, infection) or internal (for example, metabolic derangement, cytokines) triggers. Vascular injury can be antibody mediated by aPL antibodies or via accelerated classical atherosclerosis. aPL, Anti-phospholipid antibodies; BBB, Blood brain barrier; RiboP, Ribosomal-P; NMDAR, NMDA receptor. The authors are responsible for designing the figure above.

The development of NPSLE in a specific individual depends on genetic, environmental, and hormonal factors. Despite decades of research our understanding of NPSLE remains limited; however, several pathogenic pathways were identified and linked to specific clinical manifestations such as antibody-mediated neurotoxicity, vasculopathy due to anti-phospholipid (aPL) antibodies and other mechanisms, cytokine-induced neurotoxicity, and loss of neuroplasticity (Figure 1).

### Vasculopathy

While only a minority of NPSLE patients have evidence of frank vasculitis on imaging or histopathology, a small vessel thrombotic-vasculopathy has been the predominant histopathological abnormality in brains of NPSLE patients at autopsy [6]. This small vessel vasculopathy is usually non-inflammatory, and its correlation with clinical manifestations is not clear do date. It is presumed that the vascular damage to the CNS in NPSLE is due to anti-phospholipid syndrome-related vasculopathy or penetration of other autoantibodies through a damaged blood brain barrier (BBB), immune complex and complement activation, cardiac emboli caused by Libman-Zachs endocarditis, and other valvular abnormalities, vasculitis, or accelerated atherosclerosis.

### Autoantibodies

The fact that numerous autoantibodies are detected in SLE patients, and particularly in NPSLE, as well as the association between specific autoantibodies and certain manifestations suggest that their presence is linked directly to pathogenesis [5,7,8]. More than 20 autoantibodies have been linked to NPSLE [9].The identification of pathogenic autoantibodies may serve as a possible drug target in the future. A few are discussed below.

#### Anti-ribosomal-P antibodies

The presence of anti-ribosomal-P antibodies in NPSLE patients was first brought up by Bonfa et al. [10] and later in numerous cohorts of NPSLE patients [11]. Nevertheless, other reports have failed to confirm this relationship [12]. A recent meta-analysis suggested that anti-ribosomal-P antibodies are specifically related to psychosis in NPSLE [10]. Several studies demonstrated the ability of anti-ribosomal-P antibodies to bind neuronal antigens, penetrate neuronal cells, and inhibit protein synthesis [13-15]. Several autoantigens are suspected to interact with anti-ribosomal-P antibodies; however, these interactions are yet to be confirmed. Recently, it was demonstrated that anti-ribosomal-P may interact with neuronal surface-P antigen on the surface of hippocampal neurons, leading to neuronal apoptosis [16]. In this animal study, intravenous injected anti-ribosomal P was able to reach the hippocampus and cause memory impairment when the BBB was breached [16]. We recently demonstrated the binding to and penetration of anti-ribosomal-P antibodies into rat hippocampal and human neuronal cells [Kivity et al. submitted for publication]. Furthermore, in our studies, anti-ribosomal-P antibodies bonded to a neuroplasticity protein termed the growth associated protein-43. The binding of anti-ribosomal-P antibody to murine brain tissue was inhibited by the presence of this protein, thus suggesting that it may serve as an auto-antigen of anti-ribosomal-P antibodies in mice [Kivity et al. submitted for publication].

#### The anti-DNA/NR2 antibodies

While the presence of anti-DNA antibodies correlates with SLE clinical manifestations, especially glumerulonephritis and disease activity, its relationship to brain disease is less clear. Diamond et al*.* [17] demonstrated that anti-DNA can recognize a specific sequence (‘DWEYS’) contained in the N-methyl-D-aspartate (NMDA) receptors NR2a and NR2b. Passive transfer of anti-DNA/NR2 antibodies causes neuronal apoptosis. In addition, active immunization with the DWEYs followed by breaching of the BBB with lipopolysaccharide caused hippocampal neuron damage coupled with memory loss [18]. These anti-DNA/NR2 antibodies can be detected in the serum and cerebrospinal fluid (CSF) of 25 to 50% of SLE patients and some studies have found a correlation between their blood levels and NPSLE symptoms [17]. Patients with the severe form of diffuse-NPSLE (acute confusional state) demonstrate exceptionally high levels of anti-NR2 antibodies in the CSF accompanied by significant BBB damage [19]. Murine studies demonstrated that at low concentration, NMDA receptor-specific antibodies alter neural synaptic transmission, whereas at high concentration they induce neuronal death, this may explain why cognitive dysfunction is transient in some patients and permanent in others [20].

#### Anti-DNA/16–6 idiotype

The 16–6 idiotype (Id) was originally a monoclonal antibody directed against human single-stranded-DNA. Over the years, the 16–6 Id has been detected in up to 30% of lupus patients and was found to correlate with disease activity [21]. The anti-DNA/16–6 Id is related to NPSLE symptoms and can bind to human brain tissue *ex vivo* [21]. Intra-cerebro-ventricular injection of anti-DNA/16–6 Id was found to cause histological changes in the hippocampus and amygdala as well as behavioral and cognitive functions in mice [22].

#### Anti-phospholipid/anticardiolipin

Anti-phospholipid (aPL) autoantibodies are directed against epitopes of anionic phospholipids or phospholipid-binding proteins (for example, B2Gp1). These antibodies are the most studied in NPSLE, yet their pathogenesis is not clear. Their net effect, however, is activation of coagulation pathways such as interference of fibrinolysis and natural anticoagulants (for example, protein C and S), endothelial cell activation, complement activation, platelet activation, and more [23]. SLE patients with secondary anti-phospholipid syndrome are prone to focal neurological manifestations such as stroke, transverse myelitis, and chorea, as well as seizures, migraine, and cognitive impairments [23].

#### Anti-GABA

A recent study demonstrated high levels of novel autoantibodies to GABA (B1 and B2) receptors in sera and CSF of NPSLE patients [24].

### Blood brain barrier (BBB) disruption

In order to enable auto-antibodies to penetrate the brain and cause their pathogenic effect, the BBB must be breached. Different environmental factors, such as infection, stress, and ischemia, mediated by inflammatory cytokines, may damage the BBB in different anatomical sites, thus further contributing to the variety of neuropsychiatric symptoms. Anti-ribosomal-P and anti-NR2 antibodies can induce the production of pro-inflammatory cytokines, such as interleukin (IL)-6 and IL-8, by monocytes or endothelial cells. These cytokines may cause inflammation of the BBB, further allowing the entrance of auto-antibodies to the brain [25,26]. Recently, it was suggested that TWEAK/Fn14 signaling has a role in compromising the integrity of the BBB in lupus [27].

### Cytokines

Cytokines, such as IL-2, IL-10, interferon (IFN)-α, and IFN-γ, are found to be elevated in the serum of NPSLE patients. Elevation of cytokines in the CSF is also detected in NPSLE, perhaps produced by infiltrating immune cells or local glial cells. The role of cytokines and chemokines as mediator of disease as well as target for therapy is yet to be determined [28].

### Murine models – a platform for NPSLE research

SLE murine models can be differentiated into genetically designed strains that develop a spontaneous lupus-like autoimmune disease or those induced by adjuvant or other methods in naïve mice. Genetically prone strains are characterized by varying proportions by autoantibody production, circulating immune complexes, complement consumption, and clinical manifestations such as glomerulonephritis. These strains include the NZB*/*NZWF1, MRL/lpr, NZM2410, and BXSB mice [29]. The most extensively studied strains are the MRL/lpr and NZB/NZW F1. The MRL/lpr strain develops a progressive lupus-like disease due to a molecular defect in the *FAS* gene, which is expressed in B and T lymphocytes. This leads to defective apoptosis, thus causing autoimmunity. The most prominent neuropsychiatric manifestation of the MRL/lpr mouse is depressive-like behavior, which can be assessed by the force swimming and anhedonia tests at 5 weeks [30]. Anti-depressants, and more interestingly, immunosuppressants were found to reduce depression-like behavior as well as the other SLE symptoms of this model [31]. However, there is less consistency regarding anxiety and cognitive impairment in the MRL/lpr mice [29,31]. MRL/lpr mice produce several autoantibodies (anti-ribosomal, anti-phospholipid, and anti-nucleosome) and cytokines in the serum or CSF that may correlate with neuropsychiatric symptoms [31]. Spontaneous brain morphological alterations are also demonstrated in this strain [32]. A role for the TWEAK/fn4 pathway in the pathogenesis of neuropsychiatric symptoms was suggested in MRL/lpr mice [33]. In addition, it is suggested that patterns of serum antibody binding to peptide microarrays (immune-signaturing) can predict and diagnose neuropsychiatric manifestations in MRL/lpr mice [34]. The NZB/NZWF1 is the F1 hybrid strain of a cross between New Zealand Black and New Zealand White mice. It is the first and most studied spontaneous SLE-like mouse model. This mice strain develops a severe lupus-like disease manifested at 5 to 6 months by hyperactive B and T cells, autoantibodies against nuclear antigens, defective clearance of immune complexes, and glomerulonephritis leading to death. A much more limited number of studies intended to evaluate NPSLE were done in this strain, especially due to the severity of clinical disease, its increased risk of developing inherited brain anomalies, and a relatively late onset disease.

### Clinical manifestations; case definitions (1–12)

The most frequent NPSLE manifestations are headaches, psychiatric disorders (depression and anxiety), and cognitive dysfunction. Neuropsychiatric symptoms can be among the earliest manifestations of SLE, and some reports suggest up to 40% of neuropsychiatric symptoms appear during the first year of SLE diagnosis [7]. Caucasian ethnicity and older age are associated with shorter time to neuropsychiatric damage according to the LUMINA study [35]. NPSLE symptoms can be a devastating manifestation of SLE, and a recent study demonstrated a standard mortality ratio of 9.5, markedly with acute confessional syndrome [36]. NPSLE-CNS case definitions are discussed below:

1. Although headaches were once considered one of most common manifestations of NPSLE, several studies, including a meta-analysis, have more recently concluded that the rate of headaches among SLE patients was not significantly higher than it was in the normal population. In addition, no particular mechanism was found to be responsible, and headaches were not associated with general SLE disease activity, treatment, or a specific autoantibody [37,38]. Therefore, it has been suggested that headaches should not be regarded as a major NPSLE symptom unless they are intractable to treatment.
2. Seizures, generalized or focal, may develop in 10 to 20% of SLE patients [39], and tend to occur early in its course [40], especially in SLE patients with African ethnicity [41]. Generalized seizures tend to relate to disease activity while focal seizures can occur at any stage of the disease. It is also crucial to exclude secondary causes for seizures.
3. The incidence of stroke and transient ischemic attacks is elevated among SLE patients [42]. Cerebrovascular disease in SLE is strongly related to the presence of aPL antibodies [43], accelerated atherosclerosis [44], cardio-embolism due to heart valvular abnormalities, and Libman-Sacks endocarditis [45].
4. Clinical evidence of demyelination in NPSLE is reported in approximately 0.3% of cases. Demyelination is one of the most poorly understood and studied NPSLE symptoms, and can be a clinically isolated syndrome, may overlap with another CNS demyelinating syndrome (for example, multiple sclerosis (MS)), be related to drugs, and, in some cases, the diagnosis can be made only after long follow-up [46]. However, it should be noted that up to 60% of NPSLE patients may have oligoclonal bands in their CSF, and evidence suggesting demyelination on imaging is not rare [5]. On the other hand, autoantibodies, such as aPL antibodies, may be detected in patients with pure MS [47].
5. Transverse myelitis prevalence in SLE is approximately 1.5%. Several studies link transverse myelitis-SLE with aPL antibodies [48], thus suggesting spinal cord necrosis due to thrombosis as an etiology. In some cases, an overlap with Devic’s syndrome with the presence of anti-NMO antibodies is suspected. In others, transverse myelitis may convert to definite MS.
6. Chorea is the most common movement disorder in SLE and appears in 2 to 3% of patients [49,50] and even in a higher percentage in children, while Parkinsonism, ataxia, and hemiballismus are relatively rare. Chorea usually presents during the first years of SLE, and is accompanied by aPL antibodies in up to 92% of cases [51,52]. It has been suggested that these antibodies cross the BBB, bind to neuronal antigens, and induce this symptom [53]. Standard brain magnetic resonance imaging (MRI) has failed to demonstrate significant changes in choreic NPSLE patients [54], while functional imaging suggested hyperactivity in the basal ganglia [49].
7. Aseptic meningitis can be a manifestation of active SLE. Other causes of aseptic meningitis, such as infections, medication, and malignancy, should be excluded.
8. Cognitive impairment is highly prevalent among lupus patients, ranging from 20 to 80% [5,55]. Cognitive impairment does not seem to be directly attributable to disease activity, disease burden, or corticosteroid therapy [56].
  The distinction between functional and organic causes is the most difficult in the case of psychiatric disorders [57].
9. Depression is the most common mood disorder in NPLSE, and its lifetime prevalence may reach 65% [58], while mania is much less common. A recent study concluded that depression in SLE is linked to several factors of which the usage of high dose prednisone (20 mg or higher) was found to be the most significant independent factor, while global disease activity was not [59]. Other contributing factors were recent SLE diagnosis, non-Asian ethnicity, cutaneous disease, and longitudinal myelitis. These results may further support the notion that, at least for some patients, SLE related depression is associated with adverse events of therapy rather than with disease activity and may encourage clinicians to reduce prednisone doses or avoid its use [59]. An association of depression and specific antibodies directed at ribosomal-P, NMDA receptor, and other neuronal epitopes have been suggested [9,60].
10. Anxiety disorders are also common and can affect up to 40% of patients. Higher anxiety and young age are risk factors for depression [61].
11. Organic psychosis can affect 2 to 11% [62,63] of SLE patients. In approximately 60% of these, it tends to be the presenting SLE-symptom [63]. SLE psychosis usually correlates with SLE activity and responds to immunosuppressive therapy. The differential diagnosis is corticosteroid-induced psychosis, and its prevalence in SLE was not found to be higher than in other autoimmune diseases [64].
12. Acute confusional state is a diffuse neurological dysfunction that manifests as a fluctuating level of consciousness and disorientation and is equivalent to the term delirium in the DSM-IV. Due to its rather vague definition, its prevalence is difficult to estimate, ranging from 0 to 7% [65].

Other CNS manifestations that are not defined as case definitions include reversible posterior leukoencephalopathy syndrome, an increasingly recognized condition in SLE that manifests with a rapid onset of headaches, hypertension, seizures, and altered mental states. Typical neuro-imaging reveals posterior cerebral white-matter hyper-intensities. Prognosis is excellent with proper blood pressure and seizure control.

#### Olfactory impairments

SLE patients, especially those with active disease or CNS manifestations have been shown to suffer from olfactory impairments [66]. Mice injected with anti-ribosomal-P antibodies intra-cerebra-ventricularily exhibit impaired olfactory function [67], as well as MRI alterations in olfactory brain regions [68].

#### Peripheral nervous system manifestations

Peripheral nervous system manifestations affect approximately 10 to 15% of NPSLE cases, and seven are considered in the 1999 ACR-NPSLE case definitions (Box 1). The majority manifest as peripheral neuropathy [69], which includes mono or poly-neuropathy, cranial-neuropathy, inflammatory demyelinating poly-radiculoneuropathy, and plexopathy. A recent finding is that 17% of SLE-related peripheral neuropathies are small-fiber neuropathy [70]. Small-fiber neuropathies can cause severe burning pain by targeting unmyelinated C fibers and thinly myelinated A fibers. The diagnosis can be supported by skin biopsy that demonstrates damage to the dorsal root ganglia and distal axons. Other peripheral nervous system manifestations are autonomic disorders and myasthenia gravis.

### Diagnosis and imaging of NPSLE

The diagnosis of NPSLE may resemble the assembly of a puzzle: a clinician should first diagnose SLE, and then exclude non-SLE inter-current illness, medication side effects, and psychosocial- or functional-related conditions. It is important to note also that the manifestations of NPSLE might overlap the neuropsychiatric manifestations of Sjögren’s syndrome and aPL syndrome as well as other autoimmune diseases. Autoantibodies are central for the diagnosis of SLE; however, note that the prevalence of anti-nuclear antibodies in healthy subjects may reach 20% at certain ages [71], and many non-SLE patients with mild CNS symptoms, such as weakness or headache, might have weakly positive anti-nuclear antibodies testing. For patients with established SLE, several autoantibodies were found to correlate with neuropsychiatric symptoms: aPL antibodies with stroke and vascular dementia, seizures, chorea, headache, and transverse myelitis; anti-ribosomal-P and depression or psychosis; anti-neuronal with cognitive dysfunction and depression; anti-ganglioside antibodies with migraine, acute confusional state, depression, and peripheral neuropathy [72]; yet, none of these antibodies can serve as a definite marker of NPSLE.

### Cerebrospinal fluid

CSF analysis in NPSLE patients may be innocent, and thus non-contributory. In some cases, such as vasculitis, aseptic meningitis, and transverse myelitis, it may have a high yield in diagnosis. Several reports demonstrated immunological markers such as anti-DNA antibodies, oligoclonal banding, immune complexes, IL-6, and markers of B-cell activation in the CSF of NPSLE patients [73-75].

### Psychological testing

Neuropsychological testing may help to differentiate between functional and non-functional disease, but these tests are prolonged and complicated and, thus, are almost never routinely performed. The 1999 ACR-NPSLE committee proposed a relatively short (1 hour) battery of neuropsychological tests for use when NPSLE is suspected (Table 1), which is shorter than the 4 to 5 hour comprehensive batteries used before. This was validated and found reliable [76].

**Table 1:** **Neuropsychological testing in neuropsychiatric lupus syndrome (**NPSLE**) - The ACR 1-hour battery proposal**

**Test**	**Evaluates**
Revised Wechsler Adult Intelligence scale	Psychomotor speed, concentration, and graphomotor abilities
Digit Symbol Substitution Test	Psychomotor speed, concentration, and graphomotor abilities
The Trail Making Test Part B	Psychomotor speed, attention, and cognitive sequencing
The Stroop Color and Word Test	Complex attention and shifting of sets by naming color print for words written in different colors
The learning trial and short delay ‘free’ scores from the California Verbal Learning Test	Learning and recall of verbal material
The immediate and 30-minute delayed-recall measure of the Rey-Osterrieth Complex Figure Test	Visual learning and memory
The Wechsler Adult Intelligence Scale	Auditory working memory
The Controlled Oral Word Association Test (Letter Fluency) and the Animal Naming Test (Category Fluency)	Letter and category verbal fluency
The Finger Tapping Test	Simple fine motor speed

Proposed 1 hour battery of neuropsychological testing in NPSLE [76].

### Biomarkers in NPSLE

In order to better screen and monitor NPSLE treatment, there is an ongoing search for biomarkers, other than autoantibodies, cytokines, and chemokines, in this patient population. Intra-thecal levels of plasminogen activator inhibitor 1 and MMP-9 were found to correlate with NPSLE activity [77]. It was recently demonstrated that the combination of blood levels of several brain-reactive proteins (neutrophil gelatinase-associated lipocalin, S100B, and S100A8/9) with anti-NR2 and anti-ribosomal-P antibody levels is associated with cognitive impairment in childhood-onset NPSLE patients [78].

### Imaging

Several imaging modalities have enhanced our ability to investigate NPSLE; others appear promising in the near future and need further research and validation.

#### Computerized tomography (CT)

CT is used mainly in emergency settings to exclude focal abnormalities such as infarcts, hemorrhage, and tumors. Chronic conditions which can be demonstrated are cortical atrophy and calcifications.

#### Magnetic resonance imaging (MRI)

MRI is widely used in NPSLE because it is sensitive, relatively available, and can exclude other neurological conditions. However, more than half of patients diagnosed with NPSLE have a normal MRI of the brain [79], this is much less so as the disease progresses and worsens. MRI is mostly sensitive to focal findings such as cerebrovascular disease and myelitis (80 to 90%), while its sensitivity decreases for white matter lesions, gray matter lesions, and cerebral atrophy [80]. It should be noted that the latter findings can be found in non-SLE neuropsychiatric patients as well.

#### Other imaging modalities

Single photon emission computed tomography (SPECT) provides an estimate of regional cerebral blood flow and neuronal integrity and was thought to be more sensitive than MRI for the evaluation of NPSLE by some researchers. However, studies were inconsistent. Positron emission tomography–CT measures radio-labeled oxygen and glucose uptake by the brain. Several studies demonstrated an alteration in cerebral metabolism in NPSLE [81]. However, this modality is expensive, difficult to perform, and has not yet been proven to significantly contribute to an NPSLE work up [33]. A few studies have shown defective brain activity during activity such as memory tasks using functional MRI. In a recent study, childhood-onset NPSLE patients with cognitive impairment, demonstrated differential activation of functional neuronal networks during functional MRI tasks, suggesting this modality can serve as an imaging biomarker [80]. Magnetic resonance spectroscopy (MRS) is a relatively new modality that can non-invasively quantify several metabolites in brain tissue (for example, *N*-acetyl aspartate) [82,83]. Several metabolites have been used to examine NPSLE, however, these changes are not specific to SLE and might be present in other progressive diseases of the brain such as Alzheimer’s disease and MS [84]. MRS is not currently recommended for NPSLE patients although, in the future, it may help monitor and diagnose NPSLE.

### Treatment options for neuropsychiatric lupus

Treatment of NPSLE may combine therapy directed at an underlying mechanism such as autoantibody mediated damage or a hyper-coagulable state, while controlling symptoms with anti-epileptic, anti-depressive, anti-neuropathy, and other medications. Currently, no randomized controlled studies have been done to verify therapies or protocols for specific NPSLE manifestations. Hence, treatment regimens are based on expert recommendations, case studies, and small controlled trials. NPSLE therapy should be individualized based on suspected mechanisms (for example, the presence of specific antibodies or evidence of thrombosis), severity of symptoms, expected morbidity, time from onset of symptoms, reversibility, response to prior therapies, and effect on quality of life. Symptomatic therapy alone may be considered for mild NPSLE, especially when further damage is not expected. For instance, depression, headaches, and recurrent seizures do not commonly represent active SLE disease, but rather associated conditions or sequel of previous event (for example, post-stroke seizures). In contrast, if severe/new onset disease is diagnosed, especially in the presence of high SLE activity, immunosuppressant and/or directed therapy are required to control the autoimmune process and avoid further damage. Evidence exists for off-label aggressive intervention for acute NPSLE such as aseptic meningitis, myelitis, neuropathy, and psychosis. In general, treatment options for these severe and acute neuro-psychiatric symptoms are similar to those utilized for other major organ involvement in SLE or for non-SLE CNS vasculitis. These include non-specific immunosuppression, specific immune modulation mainly targeted at the humoral arm of the immune system, and/or anti-coagulation. The most studied immunosuppressive modality is the use of systemic gluco-corticosteroids (GC) that may lead to a beneficial response in 60 to 75% of patients. High doses of GC are almost universally utilized, and for severe signs or symptoms, ‘pulse therapy’ with very high doses of GC (for example, IV 1 g solomedrol/day for 3 to 5 days) followed by oral therapy (for example, prednisone 1 mg/kg/day) have been extensively used. Although emotional instability, mood swings (for example, depression), disfigurement, and various other adverse events are common in SLE patients treated with GC, and may interfere with the appropriate appraisal of disease, it is still the most effective immediate therapy available.

In many moderate to severe NPSLE presentations, additional immune suppressants are required to control the disease and enable GC withdrawal. Cyclophosphamide (CYC) is probably the most used immunosuppressant for severe NPSLE. In a controlled clinical trial [85], the addition of IV CYC to methylprednisolone was superior to therapy with methylprednisolone pulses alone for 32 patients with acute severe NPSLE (refractory seizures, cranial or peripheral neuropathy, optic neuritis, transverse myelitis, brainstem disease, and coma). All patients were treated with prednisone between pulses. Due to the effects of CYC, ovarian or sperm preservation should be considered. In another study, 37 out of 60 patients with NPSLE were treated with low doses of IV CYC (200 to 400 mg per month). Treated patients had a statistically significant improvement when compared to the control group that was treated with prednisone and plaquenil only [86]. Several reports describe successful use of azathioprine and mycophenolate mofetil as second line therapy [87] and for ‘maintenance therapy’ in order to avoid prolonged exposure to high dose steroids or as a substitute for prolonged therapy with CYC. For refractory disease, and particularly NPSLE which is considered to be induced by autoantibodies, therapy with anti-B cell therapy (rituximab), plasma exchange, or intravenous immunoglobulins (IVIG) may be considered.

#### Anti-B cell therapies

B-lymphocytes play a central role in lupus pathogenesis, therefore, drugs such as anti-CD20 monoclonal antibodies (for example, Rituximab), which directly affect several B cell populations, were suggested for refractory disease. In a recent systematic review, rituximab was beneficial in 73 to 100% of 38 patients with refractory NPSLE; however, relapse rates were high [88], suggesting that repeated therapy with rituximab may be warranted. The selection and survival of B cells are controlled by a variety of signals, including those provided by the longevity factor, B cell activating factor. Belimumab is a fully human monoclonal antibody directed against B cell activating factor that has recently been proven to be a promising therapy for SLE. Note that in both randomized controlled studies with this new biological drug, patients with active NPSLE were excluded [89]. However, *post hoc* analysis performed for both phase III trials (BLISS-52 and BLISS-76) demonstrated clinical improvements in organ systems with a low prevalence (≤16%) at baseline, including the CNS [90].

Therapies directed at reducing auto-antibodies with plasmapheresis or therapy directed at specific cytokines may also be considered. Plasmapheresis, added as an adjunct therapy for chorea or myelitis in NPSLE patients has been found to be effective. In one retrospective study, plasmapheresis was added to IV CYC and GC and led to a complete remission in 54% of 10 NPSLE patients [91]. In recent years, we successfully treated several patients presenting with acute severe NPSLE with plasmapheresis. The role of IVIG for NPSLE had been studied in a small number of patients. In one study of 9 NPSLE patients with mood swings and cognitive disorders, long-term therapy with high dose IVIG was found to be beneficial and safe [92].

#### Anti-aggregation/anticoagulation therapy

There is no question that thrombosis plays a major role in the pathogenesis of NPSLE, especially in patients with aPL antibodies. This is typically indicated when manifestations are focal, and both clinical and radiographic evaluation support ischemic or thrombotic events [93]. Therefore, the current recommendation is to treat patients with SLE who are seropositive to aPL antibodies with anti-aggregants as primary prevention, while the addition of anticoagulants is usually reserved for secondary prevention [94].

Last but not least, management of precipitating factors is imperative and includes control of hypertension, infection, metabolic abnormalities, valvular disease, and adverse drug effects [93].

### Future studies

Specific anti-IL-6 drugs have been developed in the last decade and studied extensively in several autoimmune conditions. NPSLE was linked with high levels of IL-6 as well as with BBB disruption by the TWEAK/Fn14 pathway. Hence, therapies directed at these mediators may be of value also in NPSLE, although clinical studies are still lacking.

## Conclusions

During the last few decades, overwhelming efforts were made to elucidate the pathophysiology as well as to improve the classification, diagnosis, and management of NPSLE. This accumulated information has enhanced our understanding and ability to help patients. However, several main issues remain to be solved, namely should diffuse, mild, and non- specific symptoms (for example, headaches) be considered the same disease as the more established manifestations of NPSLE? It is highly unlikely that the same etiologies are responsible for all NPSLE patients; therefore, better sub-grouping is required for clinical and research purposes. What is the true role of auto-antibodies in NPSLE, and how profound is their role in the pathogenesis of these rather devastating manifestations? The role of new imaging modalities (for example, MRS and SPECT, etc.) in the diagnosis and follow-up of NPSLE should be defined. New biomarkers should be found. We suggest the development of a severity NPSLE score, based on symptoms, imaging, and laboratory results. This score will be aimed to guide treatment intensity and help monitor clinical studies. Future studies of these urgent questions are needed, especially in the current era of biological, target therapies, and personalized medicine.

## **Box 1 Nineteen case definitions for neuropsychiatric lupus syndrome**

### Central nervous system

1. Headache
2. Seizure disorders
3. Cerebrovascular disease
4. Demyelinating syndrome
5. Myelopathy
6. Movement disorder
7. Aseptic meningitis
8. Cognitive dysfunction
9. Mood disorder
10. Anxiety disorder
11. Psychosis
12. Acute confusional state

### Peripheral nervous system

1. Mononeuropathy
2. Polyneuropathy
3. Cranial neuropathy
4. Acute inflammatory demyelinating polyradiculoneuropathy (Guillain-Barré syndrome)
5. Plexopathy
6. Autonomic disorder
7. Myasthenia gravis

Case definitions are based on the 1999 American College of Rheumatology recommendations in neuropsychiatric lupus syndrome [1].

## Abbreviations

ACR: American College of Rheumatology
aPL: Anti-phospholipid
BBB: Blood brain barrier
CNS: Central nervous system
CSF: Cerebrospinal fluid
CYC: Cyclophosphamide
GC: Gluco-corticosteroids
Id: Idiotype
IL: Interleukin
IVIG: Intravenous immunoglobulins
MRI: Magnetic resonance imaging
MRS: Magnetic resonance spectroscopy
MS: Multiple sclerosis
NMDA: N-methyl-D-aspartate
NPSLE: Neuropsychiatric systemic lupus erythematous
SLE: Systemic lupus erythematous
SPECT: Single photon emission computed tomography
